# Exposure to ambient concentrations of particulate air pollution does not influence vascular function or inflammatory pathways in young healthy individuals

**DOI:** 10.1186/1743-8977-5-13

**Published:** 2008-10-06

**Authors:** Elvira V Bräuner, Peter Møller, Lars Barregard, Lars O Dragsted, Marianne Glasius, Peter Wåhlin, Peter Vinzents, Ole Raaschou-Nielsen, Steffen Loft

**Affiliations:** 1 Institute of Public Health, Department of Environmental Health, Health Science Faculty, University of Copenhagen, Denmark; 2 Department of Occupational and Environmental Medicine, Sahlgrenska University Hospital and Academy, Gothenburg, Sweden; 3 The National Food Institute, Danish Technical University and Institute of Human Nutrition, Faculty of Life Sciences, University of Copenhagen, Frederiksberg, Denmark; 4 Department of Chemistry, University of Aarhus, Aarhus, Denmark; 5 Department of Atmospheric Environment, National Environmental Research Institute, Roskilde, Denmark; 6 Eurofins Miljø A/S, Galten, Denmark; 7 Institute of Cancer Epidemiology, Danish Cancer Society, Copenhagen, Denmark

## Abstract

**Background:**

Particulate air pollution is associated with increased risk of cardiovascular events although the involved mechanisms are poorly understood. The objective of the present study was to investigate the effects of controlled exposure to ambient air fine and ultrafine particles on microvascular function and biomarkers related to inflammation, haemostasis and lipid and protein oxidation.

**Methods:**

Twenty-nine subjects participated in a randomized, two-factor crossover study with or without biking exercise for 180 minutes and with 24 hour exposure to particle rich (number concentrations, NC: 11600 ± 5600 per cm^3^, mass concentrations: 13.8 ± 7.4 μg/m^3 ^and 10.5 ± 4.8 μg/m^3 ^for PM_10-2.5 _and PM_2.5_, respectively) or particle filtered (NC: 555 ± 1053 per cm^3^) air collected above a busy street. Microvascular function was assessed non-invasively by measuring digital peripheral artery tone following arm ischemia. Biomarkers included haemoglobin, red blood cells, platelet count, coagulation factors, C-reactive protein, fibrinogen, interleukin-6, tumour necrosis factor α, lag time to copper-induced oxidation of plasma lipids and protein oxidation measured as 2-aminoadipic semialdehyde in plasma.

**Results:**

No statistically significant differences were observed on microvascular function or the biomarkers after exposure to particle rich or particle filtered air.

**Conclusion:**

This study indicates that exposure to air pollution particles at outdoor concentrations is not associated with detectable systemic inflammation, lipid or protein oxidation, altered haemostasis or microvascular function in young healthy participants.

## Background

Epidemiological studies have consistently identified particulate matter (PM) in ambient air as an important risk factor for morbidity and mortality related to cardiovascular diseases [1]. The biological mechanisms of action of PM are thought to involve altered cardiac autonomic function, endothelial dysfunction, inflammation, oxidative stress, and altered blood hypercoaguability with small particles being more potent than larger particles due to their higher surface area and reactivity [2-4]. The ultrafine particle fraction of PM with a diameter of less than 100 nm and the ability to translocate through the epithelium of terminal bronchioles and alveoli is thought to be important in relation to health effects, although the extent of translocation has been debated [5,6]. Traffic-related PM may be particularly relevant as indicated by source allocation in acute mortality studies [7], risk of cardiovascular events shortly after exposure in traffic [8] and mortality associated with a residential address close to major roads with dense traffic [9]. Abnormal endothelial function is a strong predictor of adverse cardiovascular outcomes [10] and widely recognized in patients with atherosclerosis and its risk factors [11]. Inhalation of high concentrations of diesel exhaust particles has been shown to impair two important and complementary aspects of vascular functions in healthy humans: the regulation of vascular tone and endogenous fibrinolysis [3,12] and we recently showed that filtration of indoor air PM in the residence significantly improved microvascular function (MVF) in an aged population [13]. Similarly, decreased flow or nitroglycerine mediated vasodilation has been associated with high ambient PM levels [14,15]. In other studies, ambient or personal exposure to PM was associated with increased levels C-reactive protein (CRP), amyloid A and blood coagulation [16,17], plasma viscosity [18], fibrinogen [19,20], increased counts of neutrophils and platelets [21,22], expression of adhesion molecules on leukocyte or in plasma [23,16,24] and the oxidation of proteins and lipids in plasma or excreted in urine [17,25]. Animal experiments also indicate that pulmonary exposure to PM causes microvascular dysfunction and inflammatory responses and that ultrafine particles elicit particularly strong responses [26-28]. However, most of the mechanistic evidence comes from experimental human or animal studies with high levels of exposure or from panel studies with associated difficulties in exposure assessment and incomplete control of confounders that hamper the establishment of causality.

The primary aim of this study was to use carefully controlled exposure to real-life ambient air particles compared with particle filtered air to address the effects of particle exposure on MVF in young and healthy non-smokers. This exposure was previously shown to cause oxidative stress-induced damage to DNA in peripheral blood mononuclear cells in the same volunteers [29]. Exercise was included in the study in order to mimic real life exposure because it increases the dose by increased ventilation rate [30]. Changes in hyperaemia induced peripheral artery tonometry (RH-PAT) were used to assess MVF. Biomarkers included haematological parameters, markers of inflammation responses (serum interleukin-6 (IL-6), tumour necrosis factor α (TNF-α), fibrinogen and CRP) and haemostasis (platelet counts and coagulation factors), as well as resistance to lipid oxidation and protein oxidation in plasma in order to elucidate potential mechanisms of action. These markers are related to the pathogenesis of atherosclerosis and associations with air pollution have been found in some studies [3,31,16,33,20,17].

## Methods

### Study design and population

Study design and recruitment methods have previously been described in detail [29]. Briefly, the project was designed as a single blind two-factor crossover study with randomised exposure to fine and ultrafine particles and/or cycling scenarios. Participants were recruited using a notice in both a local newspaper and at the local campus, University of Copenhagen. To avoid problems due to diurnal variation, participants entered the exposure chamber at the same time of the morning on each 24-h visit at either 7.00 or 7.30 am. We simulated two exposure scenarios by pumping either non-filtered or particle filtered air into the chambers from one of Copenhagen's busiest roads which has a traffic density of 49200 vehicles per 24-h. Distribution and number concentration of fine and ultrafine particles (6–700 nm) was monitored using a custom built differential-mobility particle sizer (DMPS) placed in the chamber as well as portable condensation particle counters (TSI 3007; TSI, St. Paul, MN, USA), which the subjects also carried when leaving the chamber for e.g. lung function test, whereas concentrations of O_3_, NO, NO_2 _and CO were continuously measured using monitors from Advanced Pollution Instrumentation, San Diego, CA, USA. Filtration order was randomised and all measurements were performed by subjects unaware of use of high efficiency particle filter (HEPA) filter or not in the period. Outdoor air was pumped directly into the chamber using a KVR-100 ventilator (230 m^3^/h, P = 100 Pa) giving a continuous air exchange. For the particle filtered air a Camfil FARR HEPA filter (226002A1; Sweden) was inserted in-line downstream of the ventilator. Each exposure period included 90 minutes of exercise on an ergometer bicycle after exposure times of 15 minutes and 7 1/2 hours. The median interval between individual exposures was 12 days. Each volunteer was allowed to visit the bathroom, kitchen and for measurements of lung function reported elsewhere [34] and the median period outside the chamber was 99 minutes during 24-h. Blood was sampled after 6 and 24 hours of exposure

The participants, 20 men, 9 women, aged 20–40 yrs (median 25 yrs), had normal lung function (baseline FEV_1_: 4.53 ± 0.8 L), no personal history of cardiovascular disease, a mean body mass index (BMI) of 23 (SD: 2.71) and were taking no medications. Each participant was his/her own control, excluding confounding by factors that are stable within an individual over time but vary between participants.

The study was approved by the local ethics committee (KF 01 255392) and in accordance with the Declaration of Helsinki and all participants gave written informed consent before inclusion.

### Microvascular function (MVF)

MVF was measured immediately before blood sampling and non-invasively using PAT during reactive hyperaemia, as previously described in detail [35-37]. This method assesses microvascular vasodilatation evoked by the endothelial production of NO. NO production is induced by hyperaemia associated shear stress and altered hydrostatic pressure in digital arteries following the release of the cuff.

The technique uses finger-mountable pneumatic sensors (Endo-PAT 2000, Itamar Medical Ltd, Cesaria, Israel) specifically designed to continuously digitally record the arterial pulse wave amplitude. Probe pressure is generated by a computer-controlled mini-compressor, which also contains the necessary pressure transducers, signal filters and amplifiers, data storage, signal processing means, and a screen to display the signals.

Participants were instructed to fast and refrain from beverages containing alcohol and caffeine for a minimum of 5 hours prior to testing and all tests were performed in a quiet laboratory environment. A blood pressure cuff was placed above the elbow of the right arm for hyperaemia testing while the left arm served as a control. PAT finger probes were placed on the index fingers of both hands. The test consisted of three stages: baseline recording (min. 5 minutes), brachial arterial occlusion induced by inflating the cuff to 60 mm Hg above systolic pressure (exactly 5 minutes), and a post-occlusion recording of the induced reactive hyperaemia response (min. 5 minutes). Data was digitally stored as pulse wave tracings from both hands. A MVF score describing the extent of response to hyperaemia was computed using an automated algorithm. This algorithm used the average amplitude of the PAT signal during the 1-minute period beginning 90 seconds after the cuff deflation divided by the average amplitude of the PAT signal during a 3-minute period prior to the cuff inflation to describe the extent of reactive hyperaemia. To eliminate potential confounding of systemic effects of unilateral arm ischemia, this ratio was normalised to the concurrent signal from the control arm. The resulting value was further corrected for baseline signal amplitude. Determination of the reproducibility of this method has been described elsewhere [37]. Heart rate was monitored during exercise and blood pressure was measured directly before each MVF measurement.

### Biomarkers

Red blood cells (RBC), haemoglobin, platelet counts, coagulation factors (II+VII+X), CRP and fibrinogen were measured at the Department of Clinical Biochemistry, Copenhagen University Hospital as previously detailed [29]. IL-6 and TNF-α were measured with commercially available ELISA kits (R&D Systems, Abingdon, UK) at the Sahlgrenska University Hospital, Gothenburg, Sweden.

We assessed protein oxidation by the concentration of 2-aminoapidic semialdehyde in plasma proteins (PLAAS), as described previously [38]. Susceptibility to oxidation of lipoproteins in plasma was determined by ex vivo oxidation with 2,2'-azobis(2-amidinopropane) dihydrochloride (AAPH) using a fluorescent probe as previously described [39].

### Statistics

All statistical analyses were performed using SAS software (version 9.1, SAS Inst. Inc., Cary, NC). Due to four missing points for RH-PAT and 8 missing blood samples we used mixed effect model repeated measures analysis to investigate the effect of exposure to particle rich or particle filtered air on the outcome variables: MVF quantified as MVF score, haematological and inflammation biomarkers (haemoglobin, RBC, platelets, fibrinogen, coagulation factors, CRP, IL-6 and TNF-α), as well as and oxidation of plasma protein (PLAAS) and resistance of lipids to oxidation (AAPH). Participant nested in gender was included as a random factor variable to account for inter-individual variation (random intercept). Exposure in terms of presence or absence of particle filter, lengths of exposure (6 and 24 hours) as well as exercise were included as fixed categorical explanatory variables. Age was included as a continuous linear variable because the quadratic term of age had no association with endpoints. We estimated effects of exposure adjusted for possible confounding by inclusion of O_3_, CO and NO_x _as continuous variables. The distributions of MVF scores and the biomarkers were skewed and all statistical analyses were performed on the natural logarithm of these data. Correlation coefficients between all dependent variables were assessed using Spearman correlation. Significance in the differences between number concentrations of fine and ultrafine PM, and gaseous parameters (O_3_, NO, NO_x _and CO) according to the two exposure scenarios were determined by the paired t-test. The significance threshold was P < 0.05 in all analyses.

## Results

### Exposure characterisation

The chemical composition and mass of PM in the chamber air, as well as number and size distribution of PM and the concentration of gases during the two different exposure scenarios and during the corresponding period at nearby monitoring stations at a busy street and in urban background, have recently been reported[29] Exposure chamber PM mass concentrations without filtration was around 14 μg/m^3 ^and 11 μg/m^3 ^for PM_10-2.5 _and PM_2.5_, respectively, whilst the 24-h total number concentration was 500 per cm^3 ^and 12000 per cm^3 ^for particle filtered and non-filtered air, respectively (Table 1). The particle concentrations measured by the handheld TSI3007 counter were slightly higher than the DMPS based measurements without filtration whereas the difference was larger during filtration. It is our experience that especially at low particle concentrations the DMPS shows lower levels than the handheld counters, which also recorded exposure during the lung function tests outside the chambers. The filter effectively removed particles from chamber air (P < 0.01, t-test). PM mass concentrations were too low to measure during filtration. NO_x _and NO concentrations were unaffected by filtration, whereas the O_3 _concentration was significantly (P < 0.01, t-test) reduced (possibly due to reaction with the filter material) and the CO concentration significantly increased (P = 0.04, t-test). During the non-filtered air chamber scenario the levels of PM and gases resembled the composition of a mix of urban background air with penetration and mixing with busy street air. Particles with a median diameter of 57 nm were the most abundant and also represented the major part of the surface area in both indoor and outdoor (background and urban) air and the PM_2.5 _fraction was rich in sulphur consistent with substantial contributions from long-range transport [29].

**Table 1:** 24-hour averages of total number concentrations (NC) by DPMS in the chambers and handheld CPC3007 particle counters and mass of particles in the exposure chamber and at outdoor monitoring stations.

	Exposure chamber		Outdoor monitoring stations	
	No filtration	Filtration	Urban background	Busy urban street
DMPS NC (#/cm^3^)	11600 ± 5600	555 ± 1053	7100 ± 4400	24800 ± 15100
CPC3007 NC (#/cm^3^)	12200 ± 5700	1270 ± 1320	-	-
PM_10 _(μg/m^3^)	24.3 ± 9.6	-	26.4 ± 9.7	44.2 ± 13.7
PM_10-2.5 _(μg/m^3^)	13.8 ± 7.4	-	7.3 ± 3.0	19.0 ± 4.9
PM_2.5 _(μg/m^3^)	10.5 ± 4.8	-	19.0 ± 9.0	25.2 ± 12.0

Values are mean ± SD.

### Biomarkers and function tests

MVF score, haematological measurements, oxidation related to proteins and lipids and cytokines are presented in table 2 according to activity and exposure duration.

**Table 2:** Geometric means (95% CI) of all dependent variables according to exposure, time and activity

	Time of exposure	All		Cycling		Rest	
		Non-filtered	Filtered	Non-filtered	Filtered	Non-filtered	Filtered
MVF score^a^	6 h	2.17(2.04, 2.30)	2.20(2.07, 2.34)	2.18(2.01, 2.36)	2.14(1.99, 2.31)	2.15(1.95, 2.37)	2.26(2.03, 2.50)
	24 h	2.12(2.00, 2.25)	2.04(1.92, 2.16)	2.09(1.89, 2.30)	1.98(1.81, 2.15)	2.16(2.01, 2.31)	2.10(1.92, 2.29)
							
Haemoglobin (mmol/L)	6 h	8.84(8.64, 9.05)	8.86(8.67, 9.06)	8.83(8.52, 9.14)	8.78(8.51, 9.05)	8.85(8.56, 9.15)	8.95(8.67, 9.25)
	24 h	8.81(8.62, 9.01)	8.82(8.63, 9.01)	8.71(8.43, 9.00)	8.70(8.44, 8.96)	8.91(8.64, 9.20)	8.94(8.66, 9.23)
							
Red blood cells (×10^12^/L)	6 h	4.79(4.70, 4.88)	4.80(4.71, 4.88)	4.78(4.64, 4.92)	4.75(4.64, 4.87)	4.80(4.68, 4.92)	4.85(4.72, 4.98)
	24 h	4.78(4.70, 4.87)	4.78(4.70, 4.87)	4.74(4.61, 4.87)	4.73(4.61, 4.85)	4.84(4.72, 4.95)	4.84(4.71, 4.97)
							
Fibrinogen (μmol/L)	6 h	9.0(8.8, 9.3)	9.2(9.0, 9.5)	9.1(8.7, 9.5)	9.2(8.8, 9.5)	9.0(8.6, 9.4)	9.3(8.9, 9.7)
	24 h	9.2(8.9, 9.5)	9.2(8.9, 9.5)	9.3(9.0, 9.7)	9.1(8.7, 9.6)	9.1(8.6, 9.6)	9.3(8.9, 9.6)
							
Platelets (×10^9^/L)	6 h	248(234, 264)	249(236, 264)	250(228, 273)	248(230, 268)	247(227, 269)	250(230, 273)
	24 h	240(226, 254)	241(228, 255)	242(223, 263)	240(222, 259)	237(217, 259)	243(223, 264)
							
CF (II+VII+X)^b^	6 h	1.02(0.98, 1.05)	1.03(1.00, 1.06)	1.02(0.97, 1.07)	1.04(1.00, 1.09)	1.01(0.97, 1.06)	1.02(0.98, 1.07)
	24 h	1.02(0.99, 1.06)	1.03(1.00, 1.06)	1.02(0.98, 1.07)	1.02(0.98, 1.07)	1.03(0.98, 1.07)	1.04(0.99, 1.08)
							
C-reactive protein (mg/L)	6 h	1.3(1.1, 1.5)	1.5(1.3, 1.8)	1.2(1.0, 1.4)	1.5(1.2, 2.0)	1.3(1.0, 1.8)	1.5(1.1, 1.9)
	24 h	1.4(1.2, 1.7)	1.5(1.2, 1.7)	1.5(1.2, 1.9)	1.6(1.3, 2.1)	1.3(1.0, 1.8)	1.3(1.0, 1.7)
							
Interleukin-6 (ng/L)	6 h	0.83(0.72, 0.95)	0.91(0.79, 1.05)	0.89(0.73, 1.08)	1.02(0.86, 1.20)	0.77(0.63, 0.94)	0.81(0.64, 1.03)
	24 h	0.91(0.76, 1.07)	0.83(0.70, 0.98)	0.84(0.69, 1.02)	0.90(0.70, 1.16)	0.98(0.73, 1.31)	0.76(0.61, 0.96)
							
TNF-α (ng/L)^c^	6 h	1.09(1.00, 1.20)	1.14(1.05, 1.24)	1.12(0.97, 1.28)	1.20(1.06, 1.35)	1.07(0.94, 1.22)	1.08(0.96, 1.21)
	24 h	1.17(1.06, 1.28)	1.17(1.08, 1.27)	1.20(1.08, 1.34)	1.19(1.06, 1.34)	1.13(0.97, 1.33)	1.15(1.01, 1.30)
							
PLAAS (pmol/mg)^d^	6 h	41.1(39.1, 43.1)	41.7(39.6, 43.8)	41.4(38.9, 44.1)	43.9(40.9, 47.1)	40.7(37.6, 44.0)	39.5(36.7, 42.5)
	24 h	41.3(38.9, 43.9)	42.5(39.9, 45.3)	41.4(37.6, 45.6)	43.4(39.3, 48.0)	41.2(38.0, 44.8)	41.6(38.3, 45.2)
							
AAPH (lagtime min)^e^	6 h	238(225, 251)	234(223, 245)	237(218, 257)	238(222, 255)	238(222, 257)	229(215, 245)
	24 h	236(225, 247)	232(221, 243)	234(221, 249)	235(220, 250)	237(220, 255)	229(212, 246)

^a^Microvascular function: a detailed description of the score and its relation to endothelial function is provided in the methods section

^b^Coagulation factor

^c^Tumour necrosis factor α

^d^2-aminoapidic semialdehyde in plasma proteins (pmol/mg protein)

^e^Susceptibility to lipoprotein oxidation in plasma was measured as lagtime (min) of ex vivo oxidation with AAPH (2,2'-azobis(2 aminopropane)dihydrochloride)

#### Microvascular function (MVF)

Four pulse wave tracings were not recorded due to instrument failure. There was no significant association between MVF score and exercise or exposure to particle rich or particle filtered air (Table 2 and 3). The predictive effect of exposure as a categorical variable on MVF ranged from a 3.92% decrease to a 7.25% increase in MVF score (Table 3).

**Table 3:** The predictive value of the estimates (% change) with 95% confidence intervals relative to exposure to non-filtered air in mixed effects models.

Outcome variables	% change (CI)
Microvascular function score^b^	1.11 (-3.92, 7.25)
Haemoglobin (mmol/L)	-0.30 (-1.00, 1.01)
Red blood cells (×10^12^/L)	-0.20 (-1.00, 1.00)
Fibrinogen (μmol/L)	-1.10 (-2.96, 1.01)
Platelets (×10^9^/L)	-0.20 (-1.98, .02)
Coagulation factor (II+VII+X)	-1.29 (-1.98, 1.01)
C-reactive protein (mg/L)	-9.97 (-19.7, 1.01)
Interleukin-6 (ng/L)	-0.20 (-12.2, 13.9)
Tumour necrosis factor-α (ng/L)	-1.98 (-7.69,4.08)
PLAAS (pmol/mg protein)^c^	-2.08 (-7.69, 3.05)
AAPH(lagtime min)^d^	1.82 (-1.00, 4.08)

^a^Mixed model regression with subject nested in gender used as random factor and the natural logarithm of the biomarker in question included as a continuous outcome variable. All models adjusted for age, BMI, activity and time of sampling. Non-filtered air was included in the models as a categorical (yes/no) fixed effects predictor variable. Mutual adjustment with gases did not have significant effect on the significance of the main exposure variable (not shown)

^b^A detailed description of the microvascular function score and its relation to endothelial function is provided in the methods section

^c^2-aminoapidic semialdehyde in plasma proteins

^d^Susceptibility to lipoprotein oxidation in plasma was measured as lagtime (min) of ex vivo oxidation with AAPH (2,2'-azobis(2-aminopropane)dihydrochloride)

#### Biomarkers

There was no significant difference between the exposure to particle rich or particle filtered air for any of the measured biomarkers (Table 2 and 3). Haemoglobin and RBC were significantly decreased after exercise in these models.

The inclusion of gases (O_3_, NO_x _and CO) had no significant effect on these findings.

#### Correlation between biomarkers and function tests

MVF score was not correlated with any of the biomarkers included in this study (Table 4).

**Table 4:** Correlation coefficients for all day correlations among biomarkers and microvascular function score^a^

	Haemoglobin	RBC	Fibrinogen	Platelets	II+VII+X	CRP	IL-6	TNF	PLAAS^c^	AAPH^d^	BMI^e^
MVF score^b^	-0.018	-0.009	-0.059	0.014	-0.042	-0.114	0.008	-0.113	**-0.142**	0.131	0.015
Haemoglobin		**0.765**	-0.116	-**0.320**	**0.226**	-**0.253**	-0.042	0.098	-0.060	**0.327**	**0.317**
Red blood cells (RBC)			-0.062	-**0.157**	-0.021	-**0.207**	-0.007	0.050	-0.074	**0.346**	**0.190**
Fibrinogen				**0.253**	-**0.389**	**0.355**	**0.170**	0.016	0.035	-0.110	**0.286**
Platelets					-**0.458**	0.037	-0.100	-0.103	0.017	-**0.298**	**0.209**
Coagulation factor II+VII+X						-**0.308**	-0.005	-0.002	-0.010	0.096	-0.117
C-reactive protein (CRP)							**0.228**	**0.222**	0.106	**0.278**	-0.057
Interleukin (IL-6)								0.118	**0.131**	0.063	-0.022
Tumour necrosis factor (TNF)-α^g^									0.032	-**0.135**	**-0.183**
PLAAS^c^										-0.096	0.072
AAPH^d^											0.033

^a^Correlations were made on raw data. Numbers in bold depict statistically significant values (p < 0.05)

^b^MVF, microvascular function score was positively correlated with platelets (not significant, NS), IL6 (NS), BMI(NS) and AAPH (borderline significant, P = 0.05) and negatively correlated with PLAAS (significant) and the remaining biomarkers (NS)

^c^2-aminoapidic semialdehyde in plasma proteins

^d^Susceptibility to lipoprotein oxidation in plasma was measured as lagtime (min) of ex vivo oxidation with AAPH (2,2'-azobis(2-aminopropane)dihydrochloride)

^e^Body mass index

## Discussion

We studied the effects of fine and ultrafine particles from ambient air on MVF and biomarkers related to inflammation, haemostasis and lipid and protein oxidation in healthy young adults in order to give insights in the mechanisms of cardiovascular disease related to air pollution. We used a robust and powerful study design with controlled exposure comparing particle levels corresponding to urban air with virtually no exposure as well as exercise to enhance effective exposure was used. Despite our previous reported finding that the exposure was sufficient to induce oxidative damage to DNA in peripheral blood mononuclear cells within the same participants [29], no significant effect on MVF or the present biomarkers was found.

Endothelial function constitutes an independent predictor of cardiovascular events [40,41] and the clinical implications of the association between endothelial cell dysfunction and cardiac events are well established [42,43]. A recent exposure study of 30 young healthy volunteers demonstrated that inhalation of air heavily polluted with emission from a diesel engine and a PM concentration of 300 μg/m^3 ^impaired vasomotor responses to both endothelium-dependent (acetylcholine and bradykinin) and -independent (sodium nitroprusside) vasodilators [3]. Moreover, this research group also showed that endothelium-dependent vasodilatation occurring in the presence of mild systemic inflammation was persistent 24 hours after the same exposure in 15 volunteers [4]. Similarly, a reduction in flow-mediated vasodilation was associated with high ambient levels of PM levels in subjects aged 18–50 years and sitting for two hours at bus stops [15]. We have recently shown improved MVF upon removal of particles from indoor air in the homes of healthy elderly subject [13]. These results provide a mechanistic and plausible link between traffic air pollution and acute myocardial infarction. In the present study, we used ambient air PM levels of 24 μg/m^3 ^on average and the lack of response in MVF and biomarkers may be due to this relatively low concentration, but may also indicate that the blood vessels of young subjects are less sensitive to detrimental effects of air pollution exposure at realistic concentrations. This is also in accordance with the finding that flow- and nitroglycerine-mediated vasodilation was only negatively associated with urban background levels of PM_2.5 _and sulphate and/or black carbon among diabetics of a wide age range, indicating that susceptibility including diabetes and/or age is required to detect impairment of vascular reactivity at ambient levels [14]. Moreover, a recent animal experiment found decreased endothelial function assessed as decreased acetylcholine-induced and unchanged sodium nitroprusside-induced vasodilation in aortic rings after systemic administration of diesel exhaust particles to hyperlipidemic apoE knockout mice, whereas wild-type mice showed an enhanced response [44]. It should also be considered that in patients with prior myocardial infarction and overt endothelial dysfunction no further deterioration was detected despite enhanced exercise induced coronary ischemia during high level exposure to diesel exhaust [12].

We used RH-PAT to assess MVF because this functional measure reflects coronary endothelial function. The method can identify individuals with coronary endothelial dysfunction and MVF score correlates well with flow-mediated dilation and reflects the vascular function of both conduit arteries and the microvasculature [35,36,45,46]. The role of NO production has been shown by the blunted response after administration of an NO-synthase inhibitor [47]. This method has also been used in assessment of endothelial dysfunction during pre-eclampsia [48] and obstructive sleep apnoea [49] as well as improvement after administration of cocoa flavonols [50], external counter-pulsation in patients with coronary artery disorders [37] and filtration of indoor air PM in aged volunteers [13]. In the latter study, the geometric mean (95% CI) of MVF score was 1.78 (1.68, 1.89) among 42 elderly healthy volunteers and lower than in the present study, reflecting the general consensus that aging decreases MVF [13]. In a study of patients referred for coronary angiography those showing endothelial dysfunction had an average MVF score of 1.27, whilst patients without coronary endothelial dysfunction had an average MVF score of 1.78 [45]. Endothelium-independent vasodilatation assessed by the PAT signal to nitroglycerine was similar in the two groups in that study [45]. These data support the application of MVF measured by RH-PAT as a convenient non-invasive measure of coronary endothelial function. The assessment of endothelium independent vasodilatation by measuring the PAT response to administration of nitroglycerine is not likely to have contributed with further elucidation of the mechanism of PM-induced vascular effects in the present study, because there was no change in the response to flow-mediated vasodilation.

We attempted to address oxidative stress in the plasma compartment as a potential mechanism of action, and found that the particle exposure in the present study did not alter the lagtime of lipoprotein oxidation or specific protein carbonyls (PLAAS) at lysine residues in plasma. This is interesting considering that the same subjects had increased levels of oxidatively damaged DNA in peripheral blood mononuclear cells [29]. Recently, we also found increased levels of oxidative stress-induced DNA damage the morning following exposure to traffic during biking in streets as compared to biking in a laboratory environment, further supporting that ambient levels of air pollutants are sufficiently high in Copenhagen to induce systemic effects [51]. In an earlier study we found that personal 48-h exposure to PM_2.5 _was associated with oxidative damage to both DNA and protein measured as PLAAS like in the present study [52,53], whereas a similar association with lipid oxidation measured as plasma malondialdehyde cannot be compared with the present lack of effect on copper-induced lipid peroxidation. In the present study we also included exposure with similar levels of exercise to enhance exposure due to ventilation, which had a non-significant enhancing effect on the DNA damage [29]. In the results reported here, exercise significantly and independently decreased levels of haemoglobin and RBC, but had no effect on the other biomarkers.

We did not find any changes in haematological or blood coagulation parameters or markers of systemic inflammation related to the particle exposure. Exposure to diesel exhaust at 300 μg/m^3 ^has been associated with diminished fibrinolytic capacity, whereas the plasma concentrations of von Willebrand factor activity, prothrombin fragments, CRP and fibrinogen were unaltered and finally the effects on plasma concentrations of IL-6 and TNF-α vary from being unaltered to increased. [3,33]. In another study including 15 volunteers only fibrinogen was affected by exposure to concentrated ambient air particles at mean concentrations of 120 μg/m^3 ^[20]. A recent wood-smoke exposure study included 13 healthy subjects exposed to particles at 280 μg/m^3^. This wood-smoke exposure was significantly associated with the concentrations of serum amyloid A, a cardiovascular risk factor, as well as factor VIII in plasma and the factor VIII/von Willebrand factor ratio, whereas IL-6, TNF-α, CRP levels and fibrinogen showed no increase [17]. In some panel studies, CRP levels have been found to be associated with ambient or personal PM exposure [32,16]. Accordingly, acute phase reactants such as CRP, fibrinogen and amyloid A in plasma may respond at relatively high levels of particle exposure whereas effects on cytokine levels in plasma seem unclear. The recent finding of association between expression of adhesion molecules on leukocytes or in plasma and ambient levels of PM in observational panel studies suggest that these are promising biomarkers for experimental exposure studies [23,16,24].

It will remain challenging to compare all aspects of our results to other studies with experimental exposure of humans to particles because of the different composition including diesel exhaust [54,3], wood-smoke [17], and concentrated ambient PM_2.5 _[55] as well as large differences in exposure concentrations. In addition, there are significant variations in particle constituents between cities [56]. We used real-life urban background and traffic-generated particles, which is in contrast to the use of specific emission sources which make up a variable and sometimes small component of most urban air profiles. On the other hand this also caused day-day variation in the actual exposure in chambers which may have limited the possibility for detecting effects on the endpoints. The average 24-h exposure without air filtration in the chambers was around 12000 particles per cm^3^, which is somewhat higher than the 7000 particles per cm^3 ^we have found as average 24-h exposure by means of the same handheld particle counters (TSI3007) in 15 subjects of similar age living freely in Copenhagen [51]. Moreover, in that study we also found that more oxidative damage to DNA was induced by traffic generated particles than by the same number of particles encountered at home. In the present study the exposure chambers contained mainly traffic generated particles. Nevertheless, it cannot be excluded that the exposure occurring the days before each chamber scenario influenced our findings and it could also be argued that the main change in exposure was the effect of filtration for 24 h, which was not associated with improvement in the measured endpoints. We included exercise as a factor in order to elucidate potential interactions. Thus, our scenarios are representative of outdoor compositions making it easier to determine their contribution to cardiovascular effects and render our results more comparable with epidemiological studies which are based on exposure assessment for ambient air pollution as well as applicable for risk assessment. In earlier studies, which utilised high concentrations of exposure, extrapolation to lower concentrations is difficult, therefore these results provide important complementary data which may prove useful in risk assessment.

## Conclusion

The present study of healthy, young non-smokers found no change in MVF or biomarkers of inflammation, haemostasis and oxidative stress to protein and lipids in plasma comparing controlled exposure to particle filtered air and realistic particle concentrations corresponding to ambient urban air at a level inducing oxidative damage to DNA in peripheral blood mononuclear cells. Although populations at higher risk, such as elderly or diabetics, are likely to respond differently, the present results lend no support to the notion of altered vascular function and inflammation in the cardiovascular disease pathway related to ambient air PM in a young healthy population.

## Competing interests

The authors declare that they have no competing interests.

## Authors' contributions

EVB participated in the design of the study, recruited and managed the study subjects, measured the microvascular function, performed the statistical analysis and drafted the first manuscript. PM participated in the design of the study and the data analysis. LB carried out IL-6 and TNF-α measurement. LOD carried out analysis of lipid and protein oxidation. MG and PW carried out the characterisation of the air pollutants in the exposure chambers. PV developed the exposure chambers and participated in subject management. ORN participated in the design of the study and the data analysis. SL conceived of the study, and participated in its design, coordination and data analysis. All authors read and approved the final manuscript.

## References

[B1] Miller KA, Siscovick DS, Sheppard L, Shepherd K, Sullivan JH, Anderson GL, Kaufman JD Long-term exposure to air pollution and incidence of cardiovascular events in women. N Engl J Med.

[B2] Schlesinger RB, Kunzli N, Hidy GM, Gotschi T, Jerrett M (2006). The health relevance of ambient particulate matter characteristics: coherence of toxicological and epidemiological inferences. Inhal Toxicol.

[B3] Mills NL, Tornqvist H, Robinson SD, Gonzalez M, Darnley K, MacNee W, Boon NA, Donaldson K, Blomberg A, Sandstrom T, Newby DE (2005). Diesel exhaust inhalation causes vascular dysfunction and impaired endogenous fibrinolysis. Circulation.

[B4] Tornqvist H, Mills NL, Gonzalez M, Miller MR, Robinson SD, Megson IL, MacNee W, Donaldson K, Soderberg S, Newby DE, Sandstrom T, Blomberg A (2007). Persistent endothelial dysfunction in humans after diesel exhaust inhalation. Am J Respir Crit Care Med.

[B5] Kreyling WG, Semmler-Behnke M, Moller W (2006). Ultrafine particle-lung interactions: does size matter?. J Aerosol Med.

[B6] Mills NL, Amin N, Robinson SD, Anand A, Davies J, Patel D, de la Fuente JM, Cassee FR, Boon NA, MacNee W, Millar AM, Donaldson K, Newby DE (2006). Do inhaled carbon nanoparticles translocate directly into the circulation in humans?. Am J Respir Crit Care Med.

[B7] Laden F, Neas LM, Dockery DW, Schwartz J (2000). Association of fine particulate matter from different sources with daily mortality in six U.S. cities. Environ Health Perspect.

[B8] Peters A, von KS, Heier M, Trentinaglia I, Hormann A, Wichmann HE, Lowel H (2004). Exposure to traffic and the onset of myocardial infarction. N Engl J Med.

[B9] Hoek G, Brunekreef B, Goldbohm S, Fischer P, Brandt PA van den (2002). Association between mortality and indicators of traffic-related air pollution in the Netherlands: a cohort study. Lancet.

[B10] Lerman A, Zeiher AM (2005). Endothelial function: cardiac events. Circulation.

[B11] Newby DE, McLeod AL, Uren NG, Flint L, Ludlam CA, Webb DJ, Fox KA, Boon NA (2001). Impaired coronary tissue plasminogen activator release is associated with coronary atherosclerosis and cigarette smoking: direct link between endothelial dysfunction and atherothrombosis. Circulation.

[B12] Mills NL, Tornqvist H, Gonzalez MC, Vink E, Robinson SD, Soderberg S, Boon NA, Donaldson K, Sandstrom T, Blomberg A, Newby DE (2007). Ischemic and thrombotic effects of dilute diesel-exhaust inhalation in men with coronary heart disease. N Engl J Med.

[B13] Bräuner EV, Forchhammer L, Moller P, Barregard L, Gunnarsen L, Afshari A, Wahlin P, Glasius M, Dragsted LO, Basu S, Raaschou-Nielsen O, Loft S (2008). Indoor particles affect vascular function in the aged: an air filtration-based intervention study. Am J Respir Crit Care Med.

[B14] O'Neill MS, Veves A, Zanobetti A, Sarnat JA, Gold DR, Economides PA, Horton ES, Schwartz J (2005). Diabetes enhances vulnerability to particulate air pollution-associated impairment in vascular reactivity and endothelial function. Circulation.

[B15] Dales R, Liu L, Szyszkowicz M, Dalipaj M, Willey J, Kulka R, Ruddy TD (2007). Particulate air pollution and vascular reactivity: the bus stop study. Int Arch Occup Environ Health.

[B16] Ruckerl R, Ibald-Mulli A, Koenig W, Schneider A, Woelke G, Cyrys J, Heinrich J, Marder V, Frampton M, Wichmann HE, Peters A (2006). Air pollution and markers of inflammation and coagulation in patients with coronary heart disease. Am J Respir Crit Care Med.

[B17] Barregard L, Sallsten G, Gustafson P, Andersson L, Johansson L, Basu S, Stigendal L (2006). Experimental exposure to wood-smoke particles in healthy humans: effects on markers of inflammation, coagulation, and lipid peroxidation. Inhal Toxicol.

[B18] Peters A, Doring A, Wichmann HE, Koenig W (1997). Increased plasma viscosity during an air pollution episode: a link to mortality?. Lancet.

[B19] Schwartz J (2001). Air pollution and blood markers of cardiovascular risk. Environ Health Perspect.

[B20] Ghio AJ, Hall A, Bassett MA, Cascio WE, Devlin RB (2003). Exposure to concentrated ambient air particles alters hematologic indices in humans. Inhal Toxicol.

[B21] Salvi S, Blomberg A, Rudell B, Kelly F, Sandstrom T, Holgate ST, Frew A (1999). Acute inflammatory responses in the airways and peripheral blood after short-term exposure to diesel exhaust in healthy human volunteers. Am J Respir Crit Care Med.

[B22] Salvi SS, Nordenhall C, Blomberg A, Rudell B, Pourazar J, Kelly FJ, Wilson S, Sandstrom T, Holgate ST, Frew AJ (2000). Acute exposure to diesel exhaust increases IL-8 and GRO-alpha production in healthy human airways. Am J Respir Crit Care Med.

[B23] Frampton MW, Stewart JC, Oberdorster G, Morrow PE, Chalupa D, Pietropaoli AP, Frasier LM, Speers DM, Cox C, Huang LS, Utell MJ (2006). Inhalation of ultrafine particles alters blood leukocyte expression of adhesion molecules in humans. Environ Health Perspect.

[B24] O'Neill MS, Veves A, Sarnat JA, Zanobetti A, Gold DR, Economides PA, Horton ES, Schwartz J (2007). Air pollution and inflammation in type 2 diabetes: a mechanism for susceptibility. Occup Environ Med.

[B25] Sørensen M, Autrup H, Møller P, Hertel O, Jensen SS, Vinzents P, Knudsen LE, Loft S (2003). Linking exposure to environmental pollutants with biological effects. Mutat Res.

[B26] Nurkiewicz TR, Porter DW, Barger M, Castranova V, Boegehold MA (2004). Particulate matter exposure impairs systemic microvascular endothelium-dependent dilation. Environ Health Perspect.

[B27] Nurkiewicz TR, Porter DW, Barger M, Millecchia L, Rao KM, Marvar PJ, Hubbs AF, Castranova V, Boegehold MA (2006). Systemic microvascular dysfunction and inflammation after pulmonary particulate matter exposure. Environ Health Perspect.

[B28] Nurkiewicz TR, Porter DW, Hubbs AF, Cumpston JL, Chen BT, Frazer DG, Castranova V (2008). Nanoparticle inhalation augments particle-dependent systemic microvascular dysfunction. Part Fibre Toxicol.

[B29] Bräuner EV, Forchhammer L, Moller P, Simonsen J, Glasius M, Wahlin P, Raaschou-Nielsen O, Loft S (2007). Exposure to ultrafine particles from ambient air and oxidative stress-induced DNA damage. Environ Health Perspect.

[B30] Daigle CC, Chalupa DC, Gibb FR, Morrow PE, Oberdorster G, Utell MJ, Frampton MW (2003). Ultrafine particle deposition in humans during rest and exercise. Inhal Toxicol.

[B31] Baccarelli A, Zanobetti A, Martinelli I, Grillo P, Hou L, Giacomini S, Bonzini M, Lanzani G, Mannucci PM, Bertazzi PA, Schwartz J (2007). Effects of exposure to air pollution on blood coagulation. J Thromb Haemost.

[B32] Riediker M, Cascio WE, Griggs TR, Herbst MC, Bromberg PA, Neas L, Williams RW, Devlin RB (2004). Particulate matter exposure in cars is associated with cardiovascular effects in healthy young men. Am J Respir Crit Care Med.

[B33] Blomberg A, Tornqvist H, Desmyter L, Deneys V, Hermans C (2005). Exposure to diesel exhaust nanoparticles does not induce blood hypercoagulability in an at-risk population. J Thromb Haemost.

[B34] Bräuner EV, Mortensen J, Moller P, Bernard A, Vinzents P, Wahlin P, Glasius M, Loft S (2008). Effects of Ambient Air Particulate Exposure on Blood-Gas Barrier Permeability and Lung Function. Inhal Toxicol.

[B35] Kuvin JT, Karas RH (2003). Clinical utility of endothelial function testing: ready for prime time?. Circulation.

[B36] Kuvin JT, Patel AR, Sliney KA, Pandian NG, Sheffy J, Schnall RP, Karas RH, Udelson JE (2003). Assessment of peripheral vascular endothelial function with finger arterial pulse wave amplitude. Am Heart J.

[B37] Bonetti PO, Barsness GW, Keelan PC, Schnell TI, Pumper GM, Kuvin JT, Schnall RP, Holmes DR, Higano ST, Lerman A (2003). Enhanced external counterpulsation improves endothelial function in patients with symptomatic coronary artery disease. J Am Coll Cardiol.

[B38] Daneshvar B, Dragsted LO, Frandsen H, Autrup H (1997). γ-Glutamyl semialdehyde and 2-amino adipic semialdehyde: Biomarkers of oxidative damage to proteins. Biomarkers.

[B39] Young JF, Dragsted LO, Haraldsdottir J, Daneshvar B, Kall MA, Loft S, Nilsson L, Nielsen SE, Mayer B, Hyunh-Ba T, Hermetter A, Sandstrom B (2002). Green tea extract only affects markers of oxidative status postprandially: lasting antioxidant effect of flavonoid-free diet. Br J Nutr.

[B40] Halcox JP, Schenke WH, Zalos G, Mincemoyer R, Prasad A, Waclawiw MA, Nour KR, Quyyumi AA (2002). Prognostic value of coronary vascular endothelial dysfunction. Circulation.

[B41] Schachinger V, Zeiher AM (2000). Atherosclerosis-associated endothelial dysfunction. Z Kardiol.

[B42] Widlansky ME, Gokce N, Keaney JF, Vita JA (2003). The clinical implications of endothelial dysfunction. J Am Coll Cardiol.

[B43] Stocker R, Keaney JF (2005). New insights on oxidative stress in the artery wall. J Thromb Haemost.

[B44] Hansen CS, Sheykhzade M, Moller P, Folkmann JK, Amtorp O, Jonassen T, Loft S (2007). Diesel exhaust particles induce endothelial dysfunction in apoE-/- mice. Toxicol Appl Pharmacol.

[B45] Bonetti PO, Pumper GM, Higano ST, Holmes DR, Kuvin JT, Lerman A (2004). Noninvasive identification of patients with early coronary atherosclerosis by assessment of digital reactive hyperemia. J Am Coll Cardiol.

[B46] Kuvin JT, Mammen A, Mooney P, sheikh-Ali AA, Karas RH (2007). Assessment of peripheral vascular endothelial function in the ambulatory setting. Vasc Med.

[B47] Nohria A, Gerhard-Herman M, Creager MA, Hurley S, Mitra D, Ganz P (2006). Role of nitric oxide in the regulation of digital pulse volume amplitude in humans. J Appl Physiol.

[B48] Yinon D, Lowenstein L, Suraya S, Beloosesky R, Zmora O, Malhotra A, Pillar G (2006). Pre-eclampsia is associated with sleep-disordered breathing and endothelial dysfunction. Eur Respir J.

[B49] Itzhaki S, Lavie L, Pillar G, Tal G, Lavie P (2005). Endothelial dysfunction in obstructive sleep apnea measured by peripheral arterial tone response in the finger to reactive hyperemia. Sleep.

[B50] Fisher ND, Hughes M, Gerhard-Herman M, Hollenberg NK (2003). Flavanol-rich cocoa induces nitric-oxide-dependent vasodilation in healthy humans. J Hypertens.

[B51] Vinzents P, Møller P, Sørensen M, Knudsen LE, Hertel O, Schibye B, Loft S (2005). Personal exposure to ultrafine particles and oxidative DNA damage. Environ Health Perspect.

[B52] Sørensen M, Dragsted LO, Hertel O, Knudsen LE, Loft S (2003). Personal PM_2.5 _exposure and markers of oxidative stress in blood. Environ Health Perspect.

[B53] Sørensen M, Autrup H, Hertel O, Wallin H, Knudsen LE, Loft S (2003). Personal exposure to PM_2.5 _in an urban environment and biomarkers of genotoxicity. Cancer Epidemiol Biomarkers Prev.

[B54] Nemmar A, Hoet PH, Dinsdale D, Vermylen J, Hoylaerts MF, Nemery B (2003). Diesel exhaust particles in lung acutely enhance experimental peripheral thrombosis. Circulation.

[B55] Brook RD, Brook JR, Urch B, Vincent R, Rajagopalan S, Silverman F (2002). Inhalation of fine particulate air pollution and ozone causes acute arterial vasoconstriction in healthy adults. Circulation.

[B56] Sioutas C, Delfino RJ, Singh M (2005). Exposure assessment for atmospheric ultrafine particles (UFPs) and implications in epidemiologic research. Environ Health Perspect.

